# A Multi-Pathology Ballistocardiogram Dataset for Cardiac Function Monitoring and Arrhythmia Assessment

**DOI:** 10.1038/s41597-025-05287-z

**Published:** 2025-06-09

**Authors:** Jing Zhan, Zhengying Li, Xiaoyan Wu, Chao Zhang, Tao Zhao, Kewei Chen, Zhibing Lu

**Affiliations:** 1 Hubei Key Laboratory of Broadband Wireless Communication and Sensor Networks, School of Information Engineering, Wuhan University of Technology, Wuhan, 430070 Hubei China; 2 National Engineering Research Center of Optical Fiber Sensing Technology and Networks, Wuhan University of Technology, Wuhan, 430070 Hubei China; 3 State Key Laboratory of Silicate Materials for Architectures, Wuhan University of Technology, Wuhan, 430070 Hubei China; 4 State Key Laboratory of Advanced Technology for Materials Synthesis and Processing, Wuhan University of Technology, Wuhan, 430070 Hubei China; 5 Department of Cardiology, Zhongnan Hospital of Wuhan University, Wuhan, 430071 Hubei China; 6 Institute of Myocardial Injury and Repair, Wuhan University, Wuhan, 430071 Hubei China

**Keywords:** Health services, Cardiovascular diseases

## Abstract

Cardiac dysfunction plays a critical role in clinical diagnostics and treatment. Although traditional methods like echocardiography and blood biomarkers are effective, their limitations highlight the need for noninvasive and continuous monitoring solutions. Ballistocardiography (BCG), which captures subtle body vibrations generated by cardiac mechanical activity, has emerged as a promising tool for remote cardiovascular monitoring. This study presents a multi-pathology BCG dataset comprising recordings from healthy participants, patients with heart failure (HF), and those with arrhythmias such as atrial fibrillation (AF), premature ventricular contractions (PVCs), and premature atrial contractions (PACs). Synchronized electrocardiogram (ECG) and M-mode echocardiography recordings are also included, providing a comprehensive overview of cardiac function under diverse physiological and pathological conditions. The dataset aims to support the development of advanced algorithms and promote clinical validation of BCG as a tool for noninvasive cardiovascular monitoring.

## Background & Summary

Accurate monitoring and early detection of cardiac dysfunction are critical for effective clinical practice, as they directly affect diagnoses, prognostic evaluations, and treatment decisions^1,2^. Although echocardiography and blood biomarkers are widely accepted clinical tools for evaluating cardiac function, their dependence on specialized equipment and professional operation may limit their practicality for remote or home-based monitoring. Therefore, these limitations underscore the need for novel approaches that enable continuous, user-friendly, and noninvasive assessment of cardiac function, particularly in out-of-hospital settings.

Ballistocardiography (BCG) has emerged as a promising noninvasive technique for monitoring hemodynamic and cardiac cycle events. By detecting subtle vibrations on the body’s surface, BCG provides valuable information on cardiac function without the need for invasive procedures. Unlike traditional electrocardiogram (ECG)-based methods that require attaching electrodes to the body—even in portable or wearable forms—BCG is entirely unobtrusive, offering greater comfort and ease of use. In addition, BCG captures both cardiac and respiratory components, enabling the detection of subtle hemodynamic changes associated with inhalation, exhalation, and blood ejection^3,4^. For example, during inhalation, negative intrathoracic pressure increases venous return, resulting in higher wave amplitudes, while exhalation reduces cardiac output and wave amplitudes^3^. These characteristics make the BCG especially useful for measuring key indicators such as heart rate, respiratory rate, and stroke volume(SV)^5–10^.

Over the past two decades, significant advancements have been made in BCG-based vital sign monitoring and cardiovascular evaluation. Initially, research focused on improving sensor technologies, advancing from basic weighing or pressure sensors to electromechanical film sensors (EMFI)^11**–**15^, which provide more sensitive detection of micro-vibrations related to cardiac cycle events. Furthermore, various analytical techniques, including wavelet transforms^4^, empirical mode decomposition^16,17^, filtering^18^, and machine learning^19^, have been extensively explored to extract key physiological metrics such as heart rate, heart rate variability, and respiratory patterns. Moreover, BCG has been combined with complementary modalities such as ECG and photoplethysmography (PPG) to derive parameters such as pulse arrival time (PAT) and pulse transit time (PTT)^20**–**24^, helping to estimate blood pressure and providing a more comprehensive hemodynamic analysis. In addition, the feasibility of using BCG to measure advanced parameters such as cardiac output(CO)^25**–**28^ and SV^29^ has been validated, with high correlations reported with reference methods^25^. These developments suggest that BCG is likely to become a practical, noninvasive tool for long-term cardiovascular monitoring in both clinical and home settings.

In addition to its capability for quantifying hemodynamic parameters, BCG has shown increasing promise in the detection and monitoring of a variety of cardiovascular diseases, including coronary heart disease^30,31^, arrhythmias^32,33^, hypertension^34,35^, and heart failure(HF)^36**–**38^. These studies demonstrate that BCG signals can capture disease-relevant physiological changes, supporting their potential clinical utility. However, in certain areas such as valvular disease, most prior research including foundational studies from the mid-20th century^39,40^, was limited by the sensing and analysis technologies of that time. Modern validation studies employing advanced sensors and synchronized imaging are still rare, primarily due to the lack of comprehensive, publicly available datasets. Meanwhile, recent work has explored the use of BCG for sleep monitoring and broader cardiovascular risk prediction^41,42^, yet these datasets are often restricted to healthy participants or lack paired clinical imaging, which limits their usefulness in diagnostic model development.

To address these challenges, we present a comprehensive multi-pathology BCG dataset, which includes data from healthy participants as well as those diagnosed with HF, atrial fibrillation (AF), premature ventricular contractions (PVCs), and premature atrial contractions (PACs). The dataset also contains synchronized ECG and M-mode echocardiographic recordings to provide an integrated representation of cardiac function under diverse physiological and pathological conditions. M-mode echocardiography captures the motion of cardiac structures along a single ultrasound line over time, providing high temporal resolution for evaluating wall motion and chamber dimensions. It is widely used to assess left ventricular function and estimate ejection fraction(EF), offering key clinical indicators for diagnosing systolic dysfunction. Furthermore, our dataset includes left ventricular SV and EF values for all participants, measured via echocardiography at the time of signal acquisition. The SV values, combined with recorded heart rates, enable CO estimation and provide ground truth for future regression modeling. Although this study does not include direct CO analysis, the dataset is designed to support such research. In addition, EF values can be used to assess over all cardiac function.

This dataset is intended to promote research into innovative methods for assessing cardiac function, particularly in arrhythmic conditions, and to help create more accurate, cost-effective, and accessible monitoring methods for both clinical and home settings.

## Method

### Participants and ethical approval

This study enrolled 85 participants, including individuals with sinus rhythm, HF, and cardiac arrhythmias such as AF, PVCs, and PACs. Recruitment was conducted at Wuhan University of Technology and the Department of Cardiology at Zhongnan Hospital of Wuhan University between July and August 2022. Posters were displayed to invite healthy participants and those with diagnosed arrhythmias or HF. The study was approved by the Institutional Review Board of Zhongnan Hospital of Wuhan University (Ethics Approval Number: 2022075). All procedures complied with ethical guidelines, and written informed consent was obtained from all participants.

The demographic and clinical characteristics of the participants are presented in Table 1. The study population consisted of 67 participants with sinus rhythm, 15 with arrhythmias, and 7 with HF. Of these, 4 participants had both arrhythmias and HF, resulting in a total cohort of 85 unique participants. Overall, 72% of the total participants being male. The mean age of the sinus rhythm group was 42 years, whereas the arrhythmia and HF groups were older, with mean ages of 63 years. The HF group exhibited the lowest EF(%), with a mean of 39%, significantly lower than that of the sinus rhythm group (64%) and arrhythmia group (57%). Additional details, including heart rate (HR), body mass index (BMI), height (cm), and weight (kg), are provided in Table 1.

**Table 1:** Demographic and clinical characteristics of participants.

Variable	All (n = 85 Male:72%)		Sinus (n = 67, Male:75%)		Arrhythmia (n = 15, Male:73%)		HF (n = 7, Male:57%)	
	Mean(SD)	Range	Mean(SD)	Range	Mean(SD)	Range	Mean(SD)	Range
Age (years)	47 (18)	[14,91]	42 (16)	[14,74]	63 (14)	[32,91]	63 (18)	[45,91]
Height (cm)	168 (9)	[145,189]	169 (8)	[152,183]	166 (11)	[145,189]	162 (14)	[145,182]
Weight (Kg)	68 (14)	[40,115]	69 (13)	[44,115]	62 (16)	[40,98]	63 (19)	[40,94]
BMI	24 (4)	[17,40]	24 (4)	[17,40]	22 (3)	[18,27]	23 (4)	[17,28]
HR (bpm)	71 (14)	[49,119]	70 (12)	[14,74]	69 (14)	[49,93]	85 (21)	[60,115]
EF (%)	62 (9)	[34,79]	64 (6)	[52,79]	57 (14)	[34,79]	39 (5)	[34,47]

Summary of demographic and physiological characteristics for all participants (n = 85) and subgroups: sinus rhythm (n = 67), arrhythmia (n = 15), and heart failure (HF, n = 7). Data are presented as mean (SD) with corresponding range values. Age, height, weight, and BMI represent general anthropometric parameters, while ejection fraction (EF, %) serves as key cardiac function indicator.

### Inclusion and exclusion criteria

Eligible participants were screened by licensed cardiologists using echocardiography and ECG to confirm their status and ensure they met the inclusion criteria. To maintain the quality of the BCG signal, participants with severe respiratory conditions or diseases that cause involuntary body movements (e.g., tremor or muscle spasms) were excluded, as such conditions could introduce motion artifacts. Although BCG were collected in a supine position, this posture sometimes compromised echocardiographic image quality. Therefore, cases with poor quality ultrasound images were excluded to ensure the final dataset contained only clinically reliable measurements.

### Data collection requirements

**Fig. 1:**
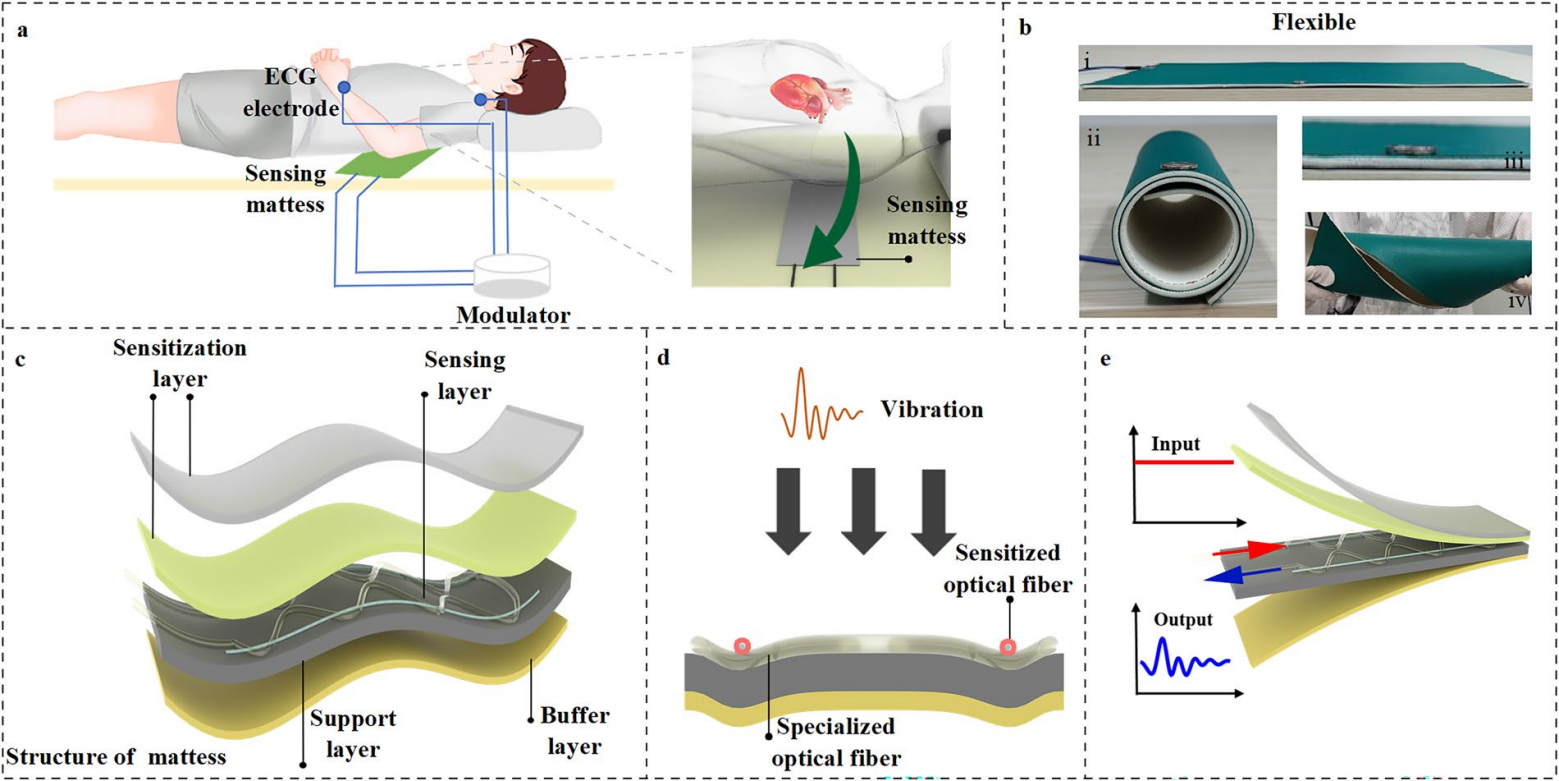
Schematic diagram of the ballistocardiogram (BCG) sensing system and sensor structure. (**a**) System setup. BCG and electrocardiogram (ECG) signals are recorded simultaneously using a sensing mattress with an embedded sensor. (**b**) Sensor flexibility. (i–iv) Demonstrations of the sensor’s flexibility and its integrated module. (**c**) Structural layers. The mattress consists of a sensitization layer (including two parts: the top is a specially hardened polyvinyl chloride material, and the second is a high-density sponge; both components are designed to enhance sensing sensitivity), a sensing layer, a support layer, and a buffer layer. (**d**) Working principle. The sensitized optical fiber deforms in response to cardiac-induced vibrations. (**e**) Signal transmission. Mechanical input modulates the optical signal, producing the processed BCG waveform.

During data collection, participants were asked to remain calm and lie flat on an optical fiber sensor mat^43^. ECG electrodes were placed on their wrist and near the collarbones to continuously monitor their heart rate as reference signals (Fig. 1a). Simultaneously, a licensed cardiologist performed M-mode echocardiographic imaging using a Mindray M7 Expert ultrasound system with a P4-2S probe, operating at a frequency of 3.2 MHz. This ensured that image acquisition adhered to clinical standards and anatomical accuracy. This integrated protocol enabled synchronized acquisition of BCG, ECG, and echocardiographic signals under well-controlled conditions, enabling accurate correlation and validation across modalities.

### Data acquisition

#### BCG recording

An optical fiber sensor system^43^ (Fig. 1a), operating at 100 Hz, was used to record BCG signals. The sensor, composed of multiple flexible layers, was placed beneath the participant to ensure comfort and adaptability (Fig. 1b,c). Its soft and pliable material allows it to bend and conform to body contours, making it ideal for long-term monitoring.

The BCG acquisition process relies on modulation and demodulation techniques within a unified system (Fig. 1e)^43,44^.When a participant lies on the sensor, heartbeat-induced micro-movements cause slight variations in the pressure exerted on the optical fiber (Fig. 1d). These variations modulate the optical signal, which is then converted into an electrical signal through demodulation. The electrical output undergoes hardware filtering and analog amplification, generating an original BCG signal that contains inherent noise. Simultaneously, the system extracts the ECG signal from the same demodulation device, ensuring precise synchronization using a shared timing clock.

#### ECG recording

ECG signals were recorded simultaneously using a dedicated AD8232 ECG module, which provides high-gain, low-noise signal acquisition for real-time heart rate monitoring. The ECG module operated at a sampling rate of 100 Hz, ensuring synchronization with the BCG signal (Fig. 1a). To maintain accurate and stable R-wave detection, standard ECG electrodes were placed on the participant’s chest, neck, and wrists to optimize signal acquisition. The ECG module was integrated into the same demodulation system as BCG, ensuring precise timing alignment under a unified clock reference.

#### M-mode echocardiograph

To further assess cardiac mechanical activity, M-mode echocardiograms were acquired during BCG and ECG monitoring using the Mindray M7 Expert ultrasound system with a P4-2S probe. All scans were performed by a licensed cardiologist to ensure operator consistency. Since BCG acquisition requires the supine position, all echocardiographic recordings were also conducted in the supine position, rather than the conventional left lateral decubitus position, to maintain alignment between BCG and ultrasound imaging. Depending on the study protocol, ultrasound images were obtained from one of three cardiac sites: the mitral valve (referred to as EJ), the aortic valve (referred to as ZJ), or the left ventricle (referred to as XJ). The echocardiographic sequences were directly saved in AVI format, ensuring compatibility with subsequent signal analysis and multi-modal integration with the BCG and ECG recordings.

#### Enhanced Singular Value Thresholding (ESVT)-based Denoising Algorithm

**Fig. 2:**
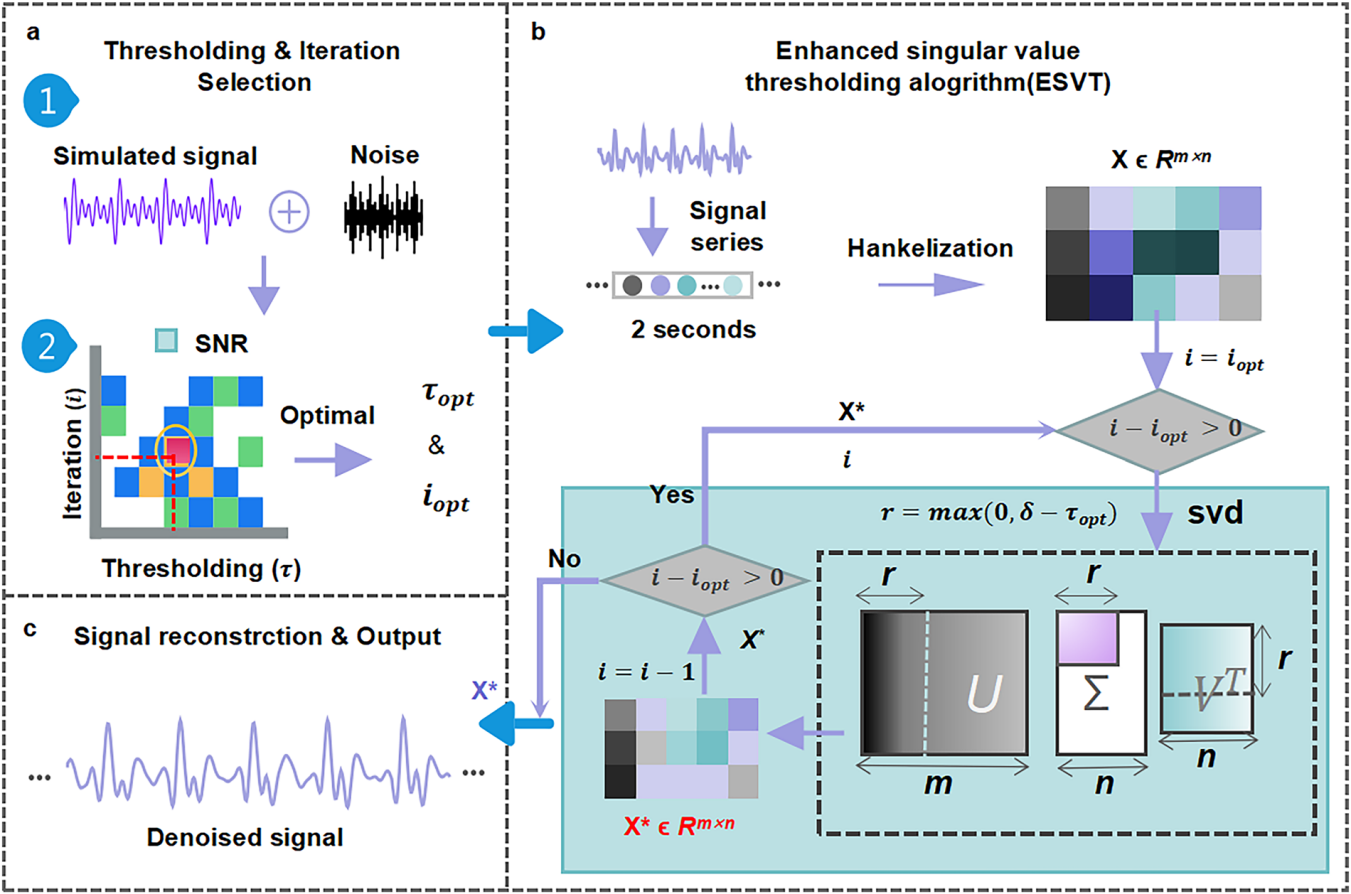
Workflow of the Enhanced Singular Value Thresholding (ESVT) algorithm. (**a**) Thresholding & iteration selection. A simulated BCG signal with Gaussian white noise undergoes a grid search to determine the optimal threshold and iteration count, based on signal-to-noise ratios(SNR) analysis. (**b**) Enhanced singular value thresholding. The noisy BCG signal is converted into a Hankel matrix, then iteratively filtered for iterations using ESVT with τ, progressively reducing noise. (**c**) Signal reconstruction and output. The denoised matrix is transformed back into the time domain, yielding a refined BCG signal with preserved physiological features.

To enhance the quality of BCG signals, we employed the Enhanced Singular Value Thresholding (ESVT) algorithm, which improves upon conventional wavelet-based denoising techniques^44,45^. The workflow of the ESVT algorithm is illustrated in Fig. 2 and consists of four main steps.

#### Simulation-guided parameter selection

To determine the optimal denoising parameters, we conducted a grid search over a range of threshold values **τ** and iteration counts ***i***.

To evaluate the performance of the proposed denoising algorithm, synthetic BCG signals were generated using a sum-of-sinusoids model informed by empirical frequency analysis. Real BCG recordings from healthy subjects were analyzed in the frequency domain, revealing five to six dominant frequency components ranging from 1.5 Hz to 9 Hz. The synthetic BCG signal was constructed as six frequency components (1.5 Hz, 3 Hz, 4.5 Hz, 6 Hz, 7.5 Hz, 9 Hz), which were combined with a low-frequency respiratory component (0.2 Hz) to simulate physiologically realistic fluctuations. The complete waveform was defined as follows:

1 $$BC{G}_{synthetic}(t)=\mathop{\sum }\limits_{k=1}^{6}{A}_{{\rm{k}}}\,\sin (2\pi {f}_{k}t)+{A}_{{\rm{r}}}\,\sin (2\pi {f}_{r}t)$$

Where ***f***_***k***_ are the dominant cardiac frequency components, and the amplitude ***A***_***k***_ set as is {0.1, 0.2, 0.1, 0.4, 0.3, 0.6}. A respiratory component at ***f***_**r**_ = 0.2 Hz with an amplitude of 2. Notably, in the actual sensing system, the analog signal is passed through a 1–30 Hz band-pass filter and then amplified by a factor of 4 before digitization. Despite this hardware filtering, residual respiratory components remain present in the recorded signals. Therefore, it is essential to include the respiratory term in the synthetic BCG waveform to accurately reflect the physiological characteristics observed in real recordings. To simulate realistic noise conditions, Gaussian white noise was added to the synthetic signal with signal-to-noise ratios (SNR) ranging from 0 to 15 dB. This choice was based on baseline noise measurements from seven unloaded sensing systems, which demonstrated that the system’s intrinsic noise followed a Gaussian distribution.

Then, a grid search was performed across different values of threshold ***τ*** and iteration count ***i***. Finally, the performance of each parameter combination was evaluated using SNR. The results were visualized as a heatmap, where the optimal parameters (***τ***_***opt***_, ***i***_***opt***_) were identified from the region with the highest SNR (deep red in Fig. 2).

#### Matrix Embedding and Singular Value Decomposition (SVD)

Once the optimal parameters were determined, the real BCG signals were processed using the following steps:

**Step 1:** The raw BCG signal ***x*(*n*)** of length ***N*** was embedded into a Hankel matrix ***X*** using a window length ***L*** (200 points, that is 2 seconds in this study).

**Step 2:** Singular Value Decomposition (SVD) was applied to decompose the matrix:

2 $${\boldsymbol{X}}={\boldsymbol{U}}{\boldsymbol{\Sigma }}{{\boldsymbol{V}}}^{{\boldsymbol{T}}}$$

where **Σ** is the diagonal matrix containing singular values **σ1** ≥ **σ2** ≥ … .

#### Thresholding process

To remove noise while preserving the primary signal components, the singular values were iteratively thresholded.

A soft-thresholding operation was applied:

3 $${{\sigma }_{{\rm{i}}}}^{{\rm{new}}}=\,\max (0,{\sigma }_{{\rm{i}}}-{\tau }_{{\rm{opt}}})$$

which selectively suppresses noise while retaining dominant periodic components.

This process was repeated for ***i***_***opt***_ iterations, ensuring progressive removal of noise components and convergence to an optimal denoised solution.

#### Signal reconstruction

The modified singular values were used to reconstruct the denoised matrix:

4 $${{\bf{X}}}^{\ast }={\bf{U}}{{\boldsymbol{\Sigma }}}^{{\bf{n}}{\bf{e}}{\bf{w}}}{{\bf{V}}}^{{\bf{T}}}$$

Then, the inverse embedding process was applied to obtain the final denoised BCG time series x*(*n*). Compared to the original signal, x*(*n*) exhibits enhanced waveform fidelity with significantly reduced noise and motion artifacts.

By iteratively refining singular value selection and thresholding, the ESVT algorithm effectively suppresses noise while preserving physiologically relevant BCG waveforms. The use of grid search-based parameter optimization ensures that the filtering process is adapted to the specific characteristics of the recorded signals, leading to improved signal quality for subsequent analysis. This structured denoising approach enhances the robustness of BCG signal processing, making it well-suited for real-world physiological monitoring applications.

## Data Records

**Fig. 3:**
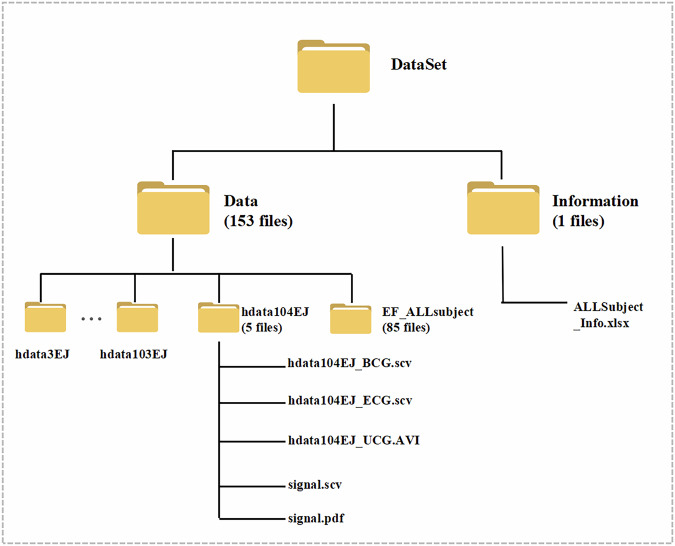
Structure of the dataset. The dataset is organized into two primary directories: *Data* and *Information*. The *Data* folder contains 153 subfolders derived from 85 participants. Among them, 152 subfolders correspond to synchronized BCG, ECG, and M-mode ultrasound recordings. Each subfolder is named using the format *hdata* + *SubjectID* + *EJ/XJ/ZJ*, where *EJ*, *XJ*, and *ZJ* indicate the echocardiographic view of the mitral valve, left ventricle, and aortic valve, respectively. The remaining subfolder, *EF_ALLsubject*, stores M-mode echocardiographic images used for ejection fraction (EF) assessment from all 85 participants. Each image file is named *hdata* + *SubjectID* + *EF* and saved in.JPG format. The *Information* folder contains demographic metadata and session-level annotations, summarized in a single master file named *Subject_Info.xlsx*, which provides subjectID, sex, age, weight, height, heart rate, EF, and available imaging types for each participant.

The dataset is available at Figshare (10.6084/m9.figshare.28416896)^46^. It is organized into two main folders: Information and Data. The data folder consists of 153 subfolders, derived from 85 participants (Fig. 3). Each participant typically contributes 2–3 subfolders, depending on the completeness and quality of their M-mode echocardiography (UCG) recordings. There are 152 subfolder named following the format: hdata + SubjectID + EJ/XJ/ZJ to denote the specific cardiac region captured in the ultrasound data: EJ denotes M-mode imaging of the mitral valve, XJ denotes M-mode imaging of the left ventricle, and ZJ denotes M-mode imaging of the aortic valve. In addition, the Data folder contains a subfolder named EF_ALLsubject, which stores EF measurement images for all 85 participants. Each image corresponds to a single participant and is named using the format hdata + SubjectID + EF. All files are saved in.JPG format, and each image shows the M-mode echocardiographic trace used for EF assessment at the time of data collection.

For instance, a participant with identifier “001” may have subfolders named hdata1EJ, hdata1XJ, and/or hdata1ZJ, corresponding to each available M-mode echocardiographic segment. Each subfolder contains five distinct files, described in detail below.

### BCG J-peak file

- File name: hdata + subjectID + EJ/XJ/ZJ_BCG.csv
- Content: J-peak positions in the BCG signal, presented in two columns.
- The first column provides the raw data point index.
- The second column specifies the corresponding time (in seconds) for each J-peak.

### ECG R-peak file

- File name: hdata + subjectID + EJ/XJ/ZJ_ECG.csv
- Content: R-peak positions in the ECG signal, also in two columns.
- The first column provides the raw data point index.
- The second column specifies the corresponding time (in seconds) for each R-peak.

### M-mode echocardiography (UCG) video

- File name: hdata + subjectID + EJ/XJ/ZJ_UCG.AVI
- Content: An AVI-format video of the simultaneously acquired M-mode echocardiogram. The suffix EJ, XJ, or ZJ indicates whether the imaging targeted the mitral valve, left ventricle, or aortic valve, respectively.

### Signal data

- File name: **signal.csv**
- Content: Three columns of time-series data sampled at 100 Hz. Raw BCG signal (Column 1).ECG data (Lead V2 or another designated lead) (Column 2). Denoised BCG signal (Column 3), derived using the ESVT algorithm.

### Signal visualization

- File name: **signal.pdf**
- Content: A graphical representation of the signals from **signal.csv**. This file facilitates quick inspection of waveform alignment and overall signal quality.

In addition to the data directory, an Information folder provides demographic and clinical details of the participants. All metadata, including date, subject IDs, demographic information, and available the type of imaging files(i.e., EJ, XJ, ZJ) with arrhythmic cases such as AF, PVCs, and PACs annotated in parentheses following the imaging type are summarized in a single Excel file named Subject_Info.xlsx, which applies to the entire dataset. Each row in **subject_info.xlsx** corresponds to an individual participant, and lists their ID, sex, weight, height, age, heart rate, and EF (%). These parameters establish an informative link between each participant’s anthropometric profile, cardiac function metrics, and the corresponding BCG, ECG, and ultrasound data.

## Technical Validation

### Parameter selection and denoising performance

**Fig. 4:**
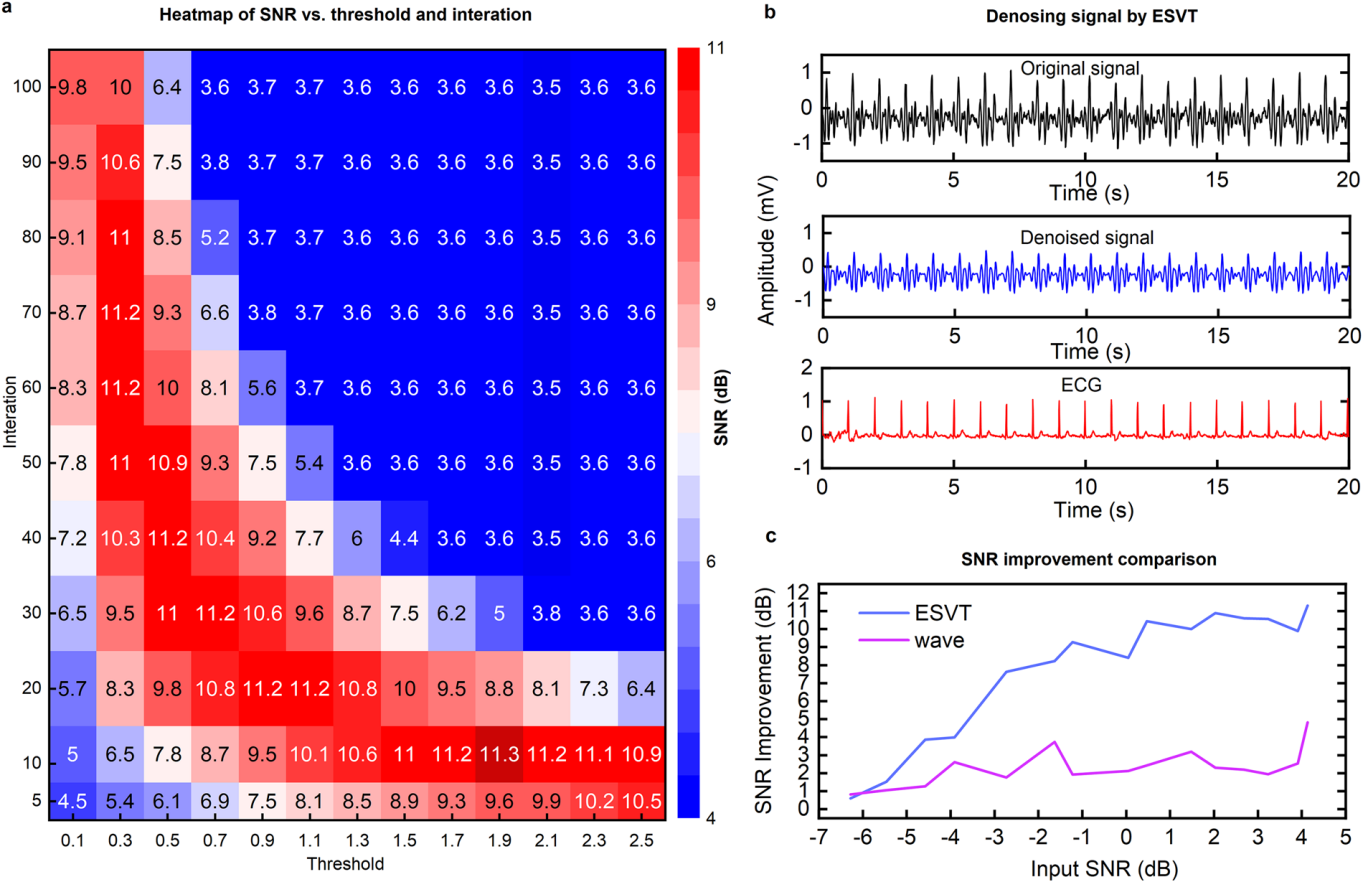
Threshold selection and denoising performance of the ESVT algorithm. (**a**) Heatmap of SNR versus threshold and iteration count. A grid search was used to identify the optimal threshold (τ_opt_) and iteration count (*i*_*opt*_), with the highest SNR values highlighted in deep red. (**b**) Denoising results of one example from a sinus cardiac cycle signal. The original noisy BCG signal (top) is processed using the optimal parameters, producing a denoised signal (middle) with improved clarity. The bottom plot shows the corresponding ECG signal for reference. (**c**) Comparison of SNR improvement between the proposed ESVT method and a conventional wavelet-based(wave) denoising technique across synthetic BCG signals. Input SNR were applied from 0 to 15 dB using Gaussian white noise. Due to the inclusion of respiratory components, the actual measured SNR ranged from approximately –6.12 dB to 4.13 dB. ESVT demonstrates superior noise suppression, particularly under low-SNR conditions.

To optimize the performance of the ESVT algorithm, we conducted a grid search over a range of threshold values (**τ**) and iteration counts (***i***). The optimal parameters were determined by evaluating the SNR, with the results visualized in a heatmap (Fig. 4a). The optimal threshold **τ**_***opt***_ and iteration count *i*_*opt*_ were selected from the region with the highest SNR values, highlighted in deep red in Fig. 4a.

Using these optimized parameters, all BCG signals in the dataset were processed. An example of a denoised BCG signal is shown in Fig. 4b, where the ESVT algorithm effectively removes high-frequency noise and low-frequency respiratory components, while preserving the primary cardiac waveform. This demonstrates that denoised BCG signals retain their physiological integrity, with minimal signal contamination, ensuring high-quality data for further analysis.

To benchmark the performance of ESVT, we compared the SNR improvement achieved by ESVT with the results from our previously uesed wavelet-based(wave) denoising approach^44^. The results (Fig. 4c) show that ESVT yields consistently higher SNR gains across all tested levels, particularly under low SNR conditions.

### Consistency between BCG-Derived and ECG-Derived Heart Rate

**Fig. 5:**
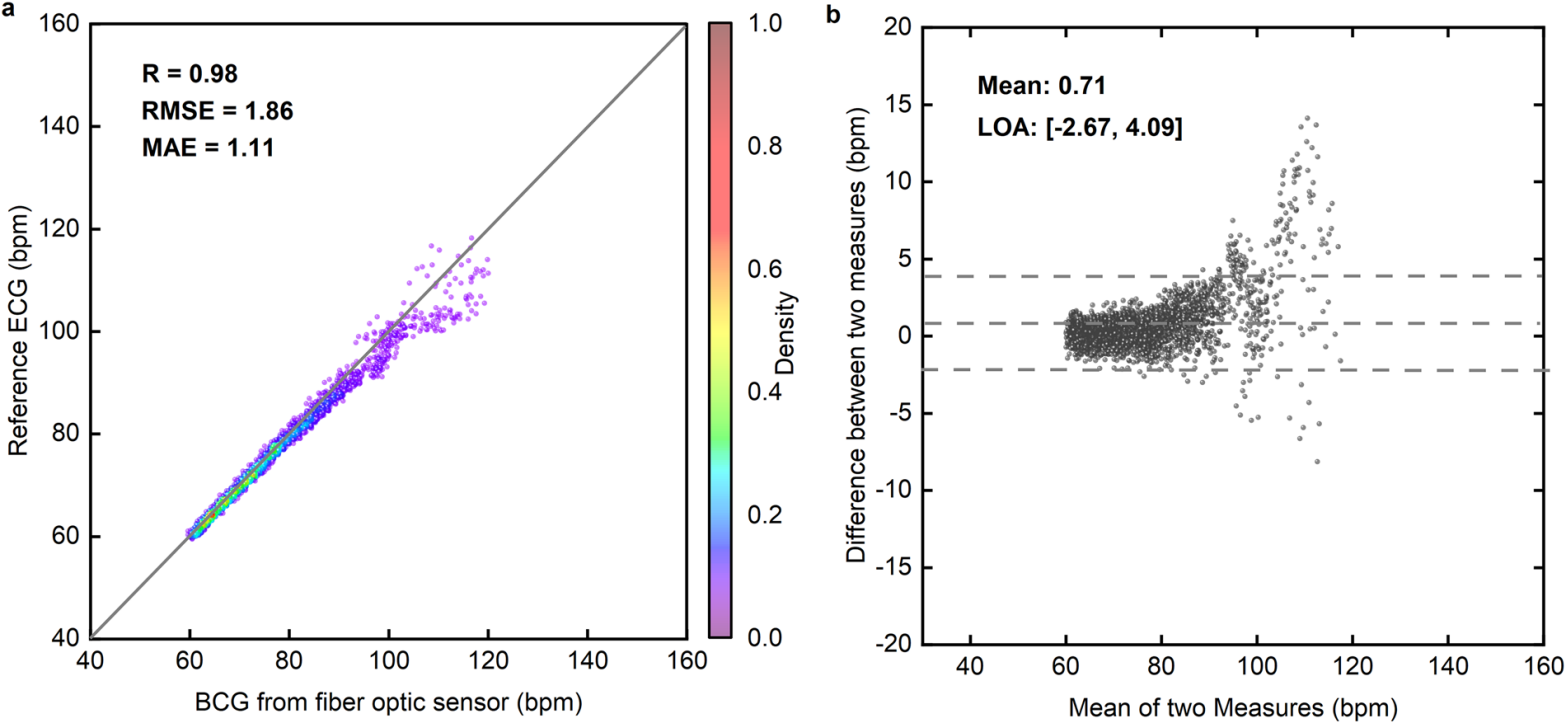
Validation of heart rate estimation from BCG signals. (**a**) Scatter plot of BCG-derived versus ECG-derived heart rates. The heart rate extracted from BCG signals is plotted against the ECG reference, with color density representing distribution. (**b**) Bland-Altman plot of heart rate differences. The differences between BCG and ECG-derived heart rates are visualized to assess agreement, with dashed lines indicating the limits of agreement (LOA).

To assess the reliability of heart rate estimation from the BCG signals of this dataset, we compared the heart rate derived from BCG with the reference heart rate from ECG in the dataset. Figure 5a presents a scatter plot of heart rates obtained from both methods, showing a strong correlation (Pearson correlation coefficient *R* = 0.98) with a low root mean square error (RMSE) of 1.86 bpm and a mean absolute error (MAE) of 1.11 bpm. The Bland-Altman plot in Fig. 5b further confirms agreement, with a mean bias of 0.71 bpm and limits of agreement (LOA) ranging from −2.67 to 4.09 bpm. These results indicate that BCG-derived heart rate measurements fall within clinically acceptable limits, aligning with established medical diagnostic standards for heart rate monitoring. This supports the feasibility of using BCG as a noninvasive alternative to ECG for clinical and ambulatory cardiac evaluations.

### Qualitative analysis of arrhythmic events

**Fig. 6:**
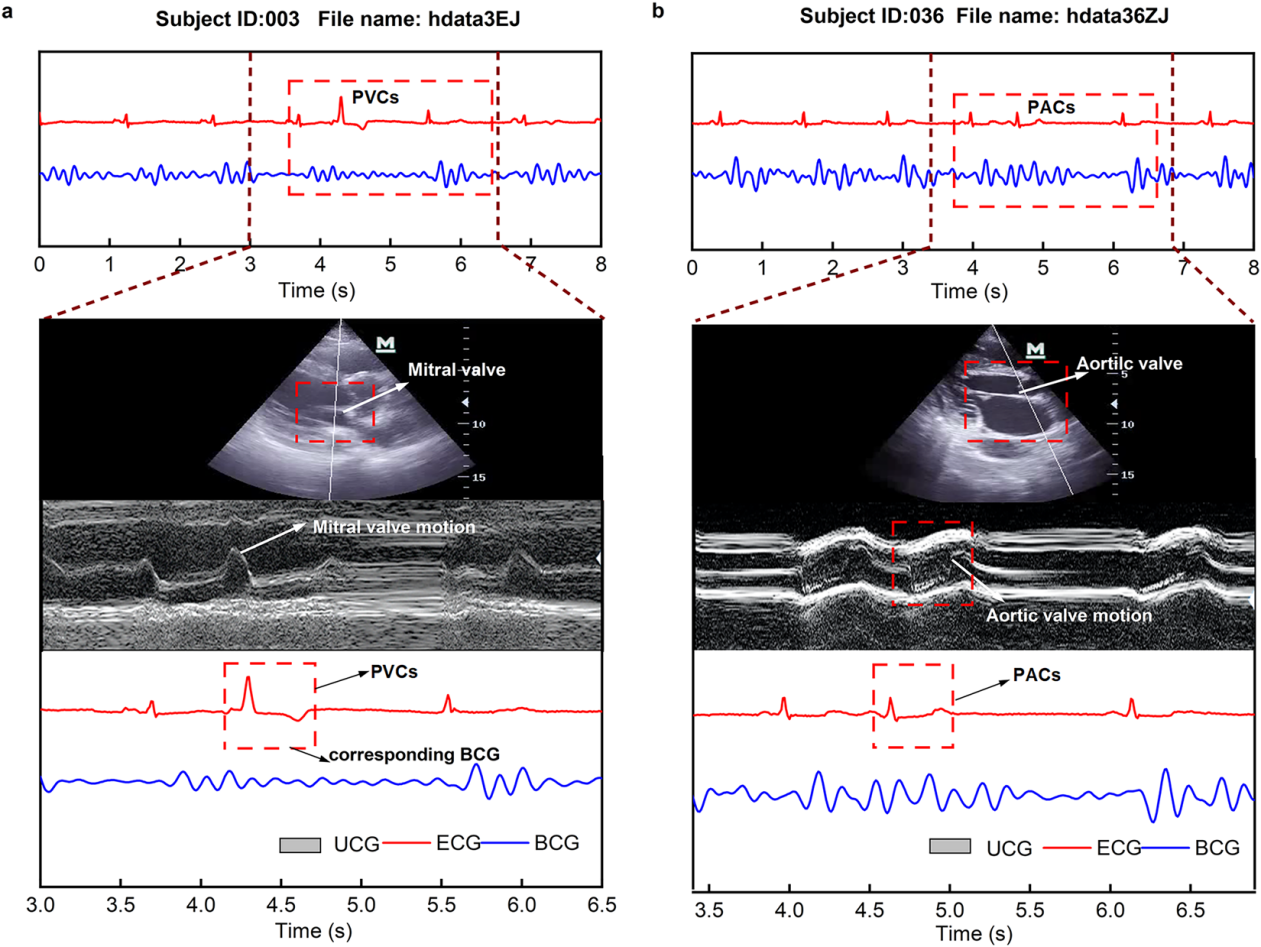
BCG and ECG signals with synchronized M-mode echocardiography in arrhythmic patients. (**a**) Mitral valve imaging (Subject ID: 003, hdata3EJ). BCG and ECG signals are shown in the upper panel, with a magnified segment highlighting an arrhythmic event. The lower panel presents the corresponding M-mode echocardiography of the mitral valve. (**b**) Aortic valve imaging (Subject ID: 036, hdata36ZJ). Similar to a, the upper panel displays BCG and ECG signals, while the lower panel shows the M-mode echocardiography of the aortic valve, synchronized with the arrhythmic episode.

A qualitative analysis was conducted on two participants(003 and 036) with cardiac arrhythmias to investigate the relationship between BCG signals, ECG, and M-mode ultrasound data (Fig. 6). In the case of the first patient with PVCs (Fig. 6a), an abnormality was observed in the ECG signal, but no corresponding mechanical response was detected in the BCG signal. Ultrasound imaging revealed that the mitral valve did not show significant movement during the PVCs event, indicating a lack of mechanical response to the abnormal electrical signal.

In contrast, for the second participant with premature atrial contractions (PACs, Fig. 6b), the BCG signal remained present and showed a slightly earlier peak than expected, consistent with the prolonged RR interval in ECG. The M-mode trace of the aortic valve captured this timing variation as well. These observations suggest that certain arrhythmic events manifest as visible changes across multiple modalities, including ECG, BCG, and ultrasound, demonstrating both temporal and morphological consistency.

To further support this, we include two additional examples (081 and 025) in the Supplementary Fig. S1 and Fig. S2, which illustrate multimodal signal alignment in additional cases of PVCs and AF, respectively. While ECG provides critical information about the heart’s electrical conduction, it does not directly reflect mechanical performance. The findings here indicate that BCG may also capture mechanical consequences of arrhythmic electrical activity, although further validation in larger cohorts is warranted.

### Dataset limitations

While the dataset includes a substantial number of high-quality BCG recordings, it currently contains a higher proportion of healthy samples compared to pathological cases. This imbalance may limit the applicability of certain machine learning models for rare or complex cardiac conditions, such as advanced heart failure or specific arrhythmia subtypes.We acknowledge this limitation and are actively working to expand the dataset by collecting and annotating more pathological BCG signals that span a broader range of clinical presentations.While BCG is not currently used as a standalone diagnostic tool in clinical settings, it holds significant potential for long-term, noninvasive monitoring of cardiac function. Future dataset releases will aim to provide a more balanced representation of subject types, thereby improving the dataset’s value for diagnostic algorithm development and validation.

In addition, we acknowledge that the left ventricular ejection fraction (LVEF) values in this dataset were measured using M-mode echocardiography. While M-mode offers high temporal resolution and ease of bedside acquisition, it has certain limitations in patients with heart failure. Specifically, M-mode relies on a single ultrasound line, and in HF cases with irregular wall motion or dilated ventricles, the positioning of the ultrasound beam may lead to underestimation of the true LVEF. In this study, we have cross-validated the M-mode EF values with the participants’ clinical diagnoses to ensure relative accuracy. Nonetheless, in future data collection efforts, we plan to incorporate B-mode echocardiographic imaging synchronized with BCG recordings to provide more comprehensive structural and functional references.

Furthermore, during comparative analysis between ECG-derived RR intervals and BCG-derived JJ intervals, we observed greater discrepancies at elevated heart rates, particularly in signals from patients with arrhythmias or heart failure. Although over 90% of cardiac cycles still exhibit clear one-to-one correspondence between ECG and BCG signals, some premature beats such as those in PVCs result in weak or absent BCG responses, thereby increasing RR–JJ divergence. We plan to investigate this phenomenon in more detail in future studies.

## Usage Notes

The dataset is available at Figshare (10.6084/m9.figshare.28416896)^46^. All files are provided in.csv,.AVI, and.pdf formats, and the data processing code is included in a separate folder labeled “code”. The data processing and analysis were performed using Python.

## Supplementary information

Supplemental information

## Data Availability

All code for data processing and technical validation are available at Figshare (10.6084/m9.figshare.28416896)^46^, and is also accessible via GitHub at: https://github.com/jingzhan1988/ESVT-algorithm-for-BCG.
