# Supplementary material for: A Multi-Pathology Ballistocardiogram Dataset for Cardiac Function Monitoring and Arrhythmia Assessment
Source: Sci Data. 2025 Jun 9;12:963. doi: 10.1038/s41597-025-05287-z (PMC12149309; doi:10.1038/s41597-025-05287-z)
Supplement: Supplementary file 1 — Supplemental information [file 41597_2025_5287_MOESM1_ESM.pdf]

A Multi-Pathology Ballistocardiogram Dataset for Cardiac Function Monitoring and Arrhythmia Assessment

Jing Zhan[1,2], Zhengying Li[1,2,3,6,*], Xiaoyan Wu[4,5], Chao Zhang[4,5], Tao Zhao[1,2], Kewei Chen[1,2], and Zhibing Lu[4,5]

[1] *Hubei Key Laboratory of Broadband Wireless Communication and Sensor Networks, School of Information Engineering, Wuhan University of Technology, Wuhan 430070, Hubei, China*

[2] *National Engineering Research Center of Optical Fiber Sensing Technology and Networks, Wuhan University of Technology, Wuhan 430070, Hubei, China*

[3] *State Key Laboratory of Silicate Materials for Architectures, Wuhan University of Technology, Wuhan 430070, Hubei, China*

[4] *Department of Cardiology, Zhongnan Hospital of Wuhan University, Wuhan, 430071, Hubei, China*

[5] *Institute of Myocardial Injury and Repair, Wuhan University, Wuhan, 430071, Hubei, China*

[6] *State Key Laboratory of Advanced Technology for Materials Synthesis and Processing, Wuhan University of Technology, Wuhan, 430070, Hubei, China*

[*]Corresponding authors: zhyli@whut.edu.cn

Contributing authors: jingzhan@whut.edu.cn, wuxiaoyan299@aliyun.com zhangchao@znhospital.cn, taozhao@whut.edu.cn,ckw@whut.edu.cn, luzhibing222@163.com

Supplementary Figures

Figure S1

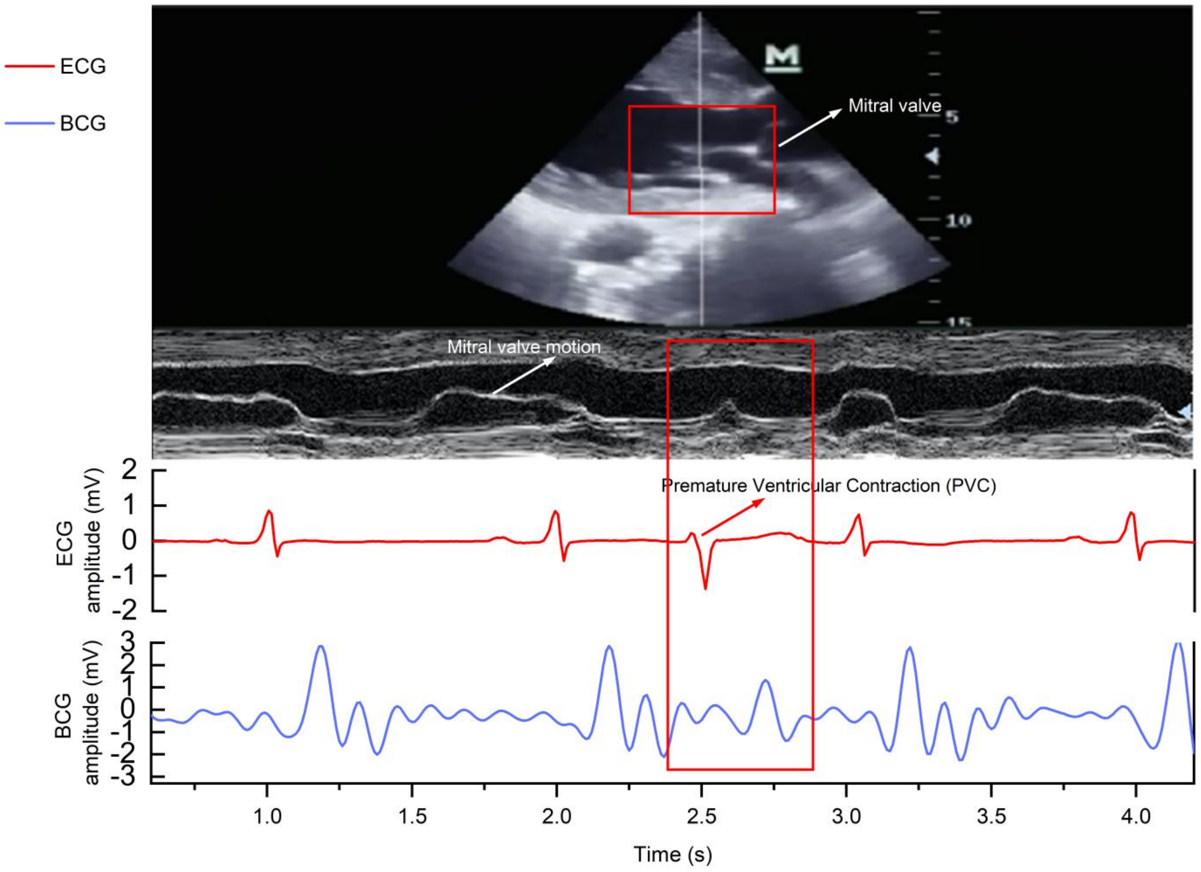

Figure S1. Synchronized recording of ECG, BCG, and M-mode echocardiography in a subject(subjectID:081) experiencing a premature ventricular contraction (PVC). The top panel shows the parasternal long-axis view with the M-mode sampling line positioned across the mitral valve. The middle panel displays the M-mode trace of mitral valve motion, where diminished displacement is observed during the PVC event (2.5–3.0 s). The bottom two plots present the synchronized ECG (red) and BCG (blue) signals.

2

Figure S2

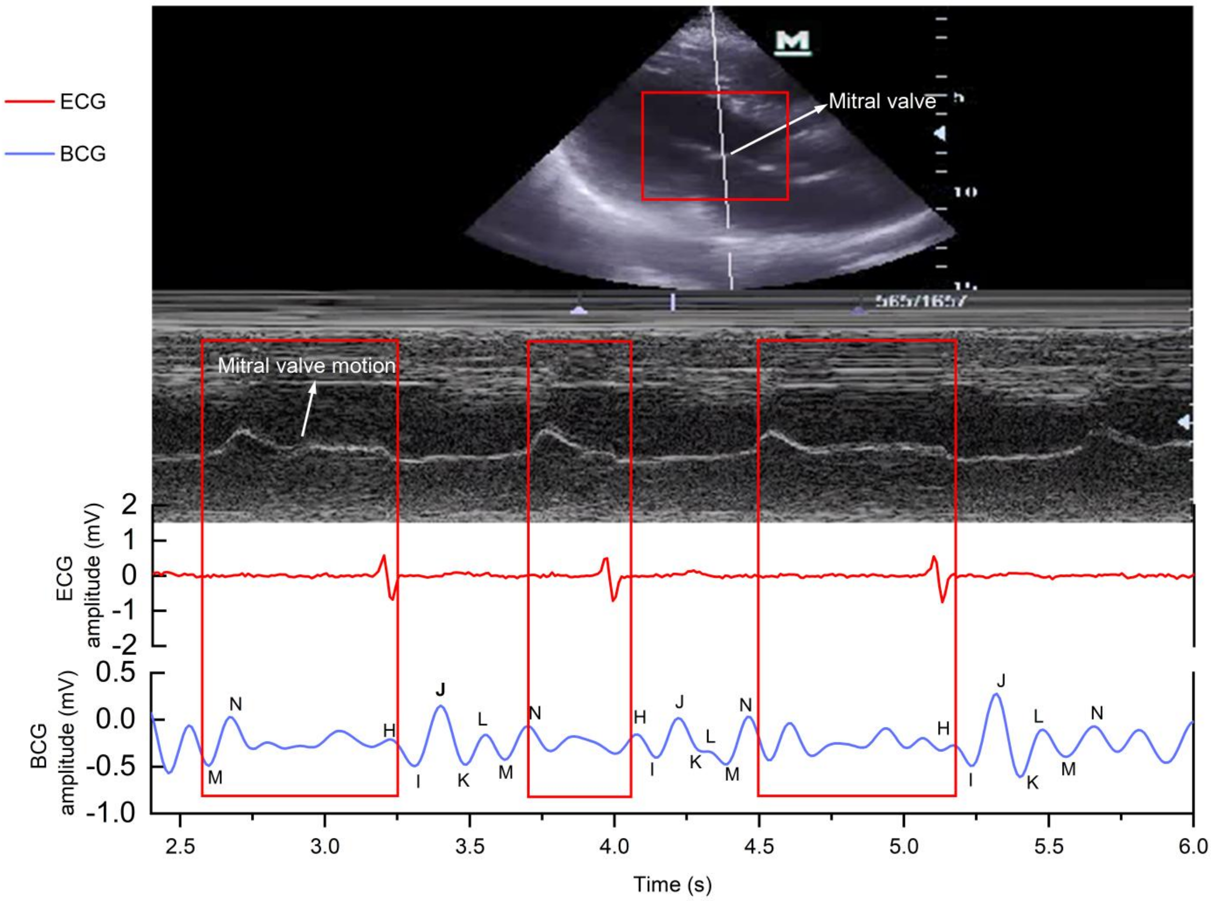

Figure S2. Synchronized ECG, BCG, and M-mode echocardiography from a subject (subjectID: 025) with atrial fibrillation (AF). The parasternal long-axis view (top panel) shows the M-mode sampling line across the mitral valve. The M-mode trace (middle panel) illustrates irregular but persistent mitral valve motion across cardiac cycles. The ECG trace (red) displays irregular RR intervals characteristic of AF, while the corresponding BCG trace (blue) reveals consistent cardiac cycle morphology with identifiable HIJKLMN waveforms in most beats. Notably, the timing of the N-to-H interval in BCG aligns well with the valve motion pattern, confirming that the BCG signal reflects cardiac mechanical activity even in the presence of arrhythmia.

3
